# What Makes Lexical Tone Special: A Reverse Accessing Model for Tonal Speech Perception

**DOI:** 10.3389/fpsyg.2019.02830

**Published:** 2019-12-18

**Authors:** Xiang Gao, Ting-Ting Yan, Ding-Lan Tang, Ting Huang, Hua Shu, Yun Nan, Yu-Xuan Zhang

**Affiliations:** State Key Laboratory of Cognitive Neuroscience and Learning, IDG/McGovern Institute for Brain Research, Beijing Normal University, Beijing, China

**Keywords:** speech perception model, lexical tone, phonological processing, tonal language, Mandarin Chinese

## Abstract

Previous studies of tonal speech perception have generally suggested harder or later access to lexical tone than segmental information, but the mechanism underlying the lexical tone disadvantage is unclear. Using a speeded discrimination paradigm free of context information, we confirmed multiple lines of evidence for the lexical tone disadvantage as well as revealed a distinctive advantage of word and atonal syllable judgments over phoneme and lexical tone judgments. The results led us to propose a Reverse Accessing Model (RAM) for tonal speech perception. The RAM is an extension of the influential TRACE model, with two additional processing levels specialized for tonal speech: lexical tone and atonal syllable. Critically, information accessing is assumed to be in reverse order of information processing, and only information at the syllable level and up is maintained active for immediate use. We tested and confirmed the predictions of the RAM on discrimination of each type of phonological component under different stimulus conditions. The current results have thus demonstrated the capability of the RAM as a general framework for tonal speech perception to provide a united account for empirical observations as well as to generate testable predictions.

## Introduction

Lexical tone is critical in determining the meaning of speech sounds for some languages such as Mandarin Chinese. For Mandarin, each spoken character (mostly monosyllable) consists of an initial consonant segment and an ending vowel segment. Lexical tone is heard on the vowel segment, but is considered a supra-segmental feature. While tonal languages have been estimated to comprise over 60% of world’ languages (Yip and Chung, 2002), speech perception studies have historically focused on non-tonal languages. How lexical tone is processed relative to and integrated with segmental information remains unclear.

Initial efforts in understanding the role of lexical tone in speech perception typically involved comparison of lexical tone with segmental information, particularly vowel segments. Evidence from a variety of tasks suggests that lexical tone information is later extracted or harder to access than segmental information (Taft and Chen, 1992; Cutler and Chen, 1997; Ye and Connine, 1999; Tong et al., 2008). For example, lexical tone-based responses have been reported to be less accurate and/or slower than those based on segmental information for homophone judgment of written characters (Taft and Chen, 1992), real word/non-word judgment (Cutler and Chen, 1997), speeded same/different discrimination (Cutler and Chen, 1997), monitoring for presence of a target element (Ye and Connine, 1999), and word reconstruction (Wiener and Turnbull, 2016). In terms of interaction between different stimulus dimensions, lexical tone judgment is also more influenced by segmental variation than vice versa (Repp and Lin, 1990; Tong et al., 2008). A tone-to-segment disadvantage has also been observed in 6-year-old Mandarin monolingual and English-Mandarin bilingual children in the form of reduced sensitivity to mispronunciations (Wewalaarachchi and Singh, 2020). On the other hand, there are reports of equal or even greater contribution of lexical tone to speech perception than segmental information. For example, lexical decision is equally accurate for tonal and segmental changes for disyllable words or idioms (Liu and Samuel, 2007). Syllable monitoring in idioms is actually faster for lexical tone than vowel (Ye and Connine, 1999). Eye movements of participants who tried to match a spoken word to pictures indicate similar contributions of lexical tone and the ending phoneme (Malins and Joanisse, 2010). Scalp-recorded potentials are also similarly modulated by semantic violations at the end of a sentence by mismatching lexical tone and vowel segment (Schirmer et al., 2005). To reconcile the discrepancy, it has been suggested that while lexical tone displays a disadvantage relative to segmental information in sub-lexical processing, its use is promoted by a constraining context *via* top-down feedback (Ye and Connine, 1999; Malins and Joanisse, 2010). However, the mechanisms underlying the sub-lexical disadvantage or the feedback advantage for lexical tone have not been specified.

Accumulating evidence suggests that perception of tone in speech involves a complicated, multi-level cortical network (Shuai and Gong, 2014; Roll et al., 2015; Si et al., 2017; Soderstrom et al., 2017; Schremm et al., 2018). For example, event-related potential (ERP) studies (Shuai and Gong, 2014) indicate temporally overlapping but spatially separate top-down, linguistic processing and bottom-up, auditory processing of lexical tones with linguistic processing lateralized toward the left hemisphere and auditory processing toward the right hemisphere. Cortical surface recordings (Si et al., 2017) further distinguished a distributed cooperative network consisting of bilateral temporal and frontal motor areas in native Chinese speakers passively listening to tones in an oddball paradigm, with strong temporal-on-motor influence in the right hemisphere. As an interesting comparison, concurrent fMRI and EEG recordings with native speakers of Swedish, in which tones on word stems are used to predict suffixes, revealed that in addition to left primary auditory cortex and superior temporal gyrus, left inferior frontal gyrus was also activated during tone perception and correlated to both early and later electrical responses (Roll et al., 2015; Soderstrom et al., 2017). The hierarchical processing structure of Swedish tone was further supported by cortical thickness measures, which correlated to tone perception in real words in left planum temporale and to tone processing in pseudo words in left inferior frontal gyrus pars opercularis (Schremm et al., 2018). Overall, these imaging studies all point to multi-level neural processing of tone related information in a complex cortical network. However, because tone is typically the only manipulated factor in such studies, a dynamic picture of processing and integration of different types of phonological information is still missing.

The increasing literature of speech perception for tonal languages calls for a theoretical framework. To date, lexical tone processing has typically been handled by simple extensions of existing speech recognition models for non-tonal languages, among which the most influential one is the TRACE model (McClelland and Elman, 1986). The TRACE is a connectionist model consisting of three levels of phonological processing: feature (such as acuteness and diffuseness), phoneme, and word, with bidirectional inhibitory connections within a level and excitatory connections between levels. Representations in TRACE subserve perception as well as working memory, in that any information, once extracted, is immediately available for mental operations. To the three levels in TRACE, Ye and Connine (1999) added a level for lexical tone, in parallel to phoneme, which they named “toneme.” To account for the reversal of or compensation for the lexical tone disadvantage brought about by a highly constraining context (Ye and Connine, 1999; Liu and Samuel, 2007; Malins and Joanisse, 2010), top-down feedback from the word level to the toneme level was assumed to be stronger than that to the phoneme level. This idea of parallel processing of lexical tone and phoneme has been computationally formulated in a recent extension of TRACE (Shuai and Malins, 2016).

**Figure 1:**
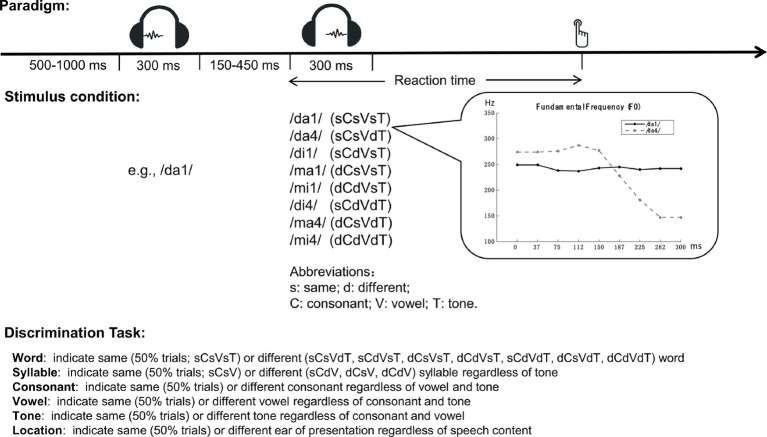
Schematic illustration of the speeded discrimination paradigm.

Here, we revisit the tone-to-segmental disadvantage, focusing on whether speech perception performance is consistent with parallel processing of lexical tone and phoneme, as assumed by simple extensions of TRACE-like models (for example, Ye and Connine, 1999; Shuai and Malins, 2016). We started with examining a variety of phenomena related to lexical tone disadvantage using the same experimental paradigm devoid of contextual information (Figure 1). Native Chinese speakers were presented with two sequential spoken monosyllable words and were asked to make speeded same/different judgments regarding the entire character or one of the phonological components: lexical tone, vowel, consonant, and atonal syllable as the combination of consonant and vowel. All tasks were performed with the same stimulus set of eight spoken words generated by independent combinations of two consonants, two vowels, and two lexical tones. Performance was measured with both response accuracy (error rate) and speed (reaction time, RT). A location task with no demand for speech information was used to provide baseline performance.

Given the current literature and the assumption of parallel processing for lexical tone and phoneme, we made two sets of predictions. First, regarding comparison between lexical tone and phoneme, we expected (1) that lexical tone judgment should be slower than vowel and consonant judgment, (2) that word judgment, a phonological decision required in homophone and lexical decision tasks, should be poorer when the stimuli differed only in lexical tone than when they differed only in segmental information, and (3) that lexical tone judgment would exhibit greater interference from phoneme variation than vice versa. Second, regarding comparison across different processing levels, we expected (1) that processing error and time should accumulate from bottom up, so that word judgment among all tasks would have the largest error rate and RT and (2) that syllable judgment should be performed at the phoneme level, rendering syllable performance rank between consonant and vowel judgments, as syllable judgment in some cases could be made as soon as consonant information is extracted and in the remaining cases should require vowel information.

Experimental results confirmed the predictions regarding tone-phoneme comparison, but defied those regarding comparisons across processing levels. The results led us to propose a novel model for sub-lexical phonological processing of Chinese speech. We further tested the model’s predictions on how each type of speech judgment should be performed in different stimulus conditions.

## Materials and Methods

### Participants and Equipment

One hundred native speakers of Mandarin Chinese (mean age: 22.8 ± 2.4 years; 61 females) were recruited from Beijing Normal University campus and gave informed consent to participation of the experiment. All of the participants had normal hearing (tone threshold ≤20 dB HL across 0.5–6 kHz at both ears) and no known cognitive or language disorders. The experimental procedure was approved by the Beijing Normal University Research Ethics Committee.

The experiment was carried out in a sound-attenuated booth using custom computer programs developed with Psychtoolbox for Matlab (Brainard, 1997; Kleiner et al., 2007). Auditory stimuli were delivered using Sennheiser HD-380 circumaural headphones.

### Behavioral Test

The same speeded discrimination paradigm and stimulus pool were used for six discrimination tasks: word, syllable, tone, consonant, vowel, and location, named after their target stimulus dimension (Figure 1). At each trial, two 300-ms spoken tokens of Chinese character (e.g., /ma1/ and /ma4/) were presented sequentially and the participants were asked to make speeded same/different judgments regarding a target stimulus dimension (e.g., lexical tone) by pressing a key on the keyboard.

For all tasks, the first token was randomly drawn from a stimulus pool and randomly presented at one of the two ears. The second token could be the same or different from the first one in location (ear of presentation), consonant, vowel, lexical tone, or their combinations. Stimuli were presented to participants at a sound level varying between 65 and 80 dB SPL across trials. The inter-stimulus interval was varied between 150 and 450 ms, and the inter-trial interval was varied between 500 and 1,000 ms. The stimulus pool consisted of eight tokens (/da1/, /da4/, /di1/, /di4/, /ma1/, /ma4/, /mi1/, and /mi4/), constructed by independently combining two consonants (/d/ and /m/), two vowels (/a/ and /i/), and two lexical tones (1 and 4, see Figure 1 inset panel for a sample of their fundamental frequency patterns). All of these tokens correspond to existent characters in modern Chinese vocabulary. For clarity, we will refer to the tonal syllable (combination of consonant, vowel, and lexical tone) as “word” and the atonal syllable (combination of consonant and vowel without lexical tone) as “syllable.” All speech stimuli were generated using the NeoSpeech Text-To-Speech engine^1^ with a female voice, at the sampling rate of 44.1 kHz.

### Stimulus Conditions

Stimulus conditions were denoted by the same/different relationship of the two tokens in consonant, vowel, and lexical tone (Figure 1). The probability of same and different in the target dimension was always equal, making chance performance to be 50% correct for all tasks. This limit rendered different distributions of trials across the eight stimulus conditions for different tasks. *For Word Discrimination*, the target dimension was word (tonal syllable: combination of consonant, vowel, and lexical tone). Difference in word was set as difference in lexical tone, syllable, or both with equal probability. Syllable difference in turn could be difference in consonant, vowel, or both with equal probability. For *Syllable and Tone Discrimination*, syllable and lexical tone relationships were independently randomized. Same as for *Word Discrimination*, syllable difference was set with equal probability as difference in consonant, vowel, or both. For *Consonant and Vowel Discrimination*, consonant, vowel, and lexical tone relationships were all independently randomized, giving rise to approximately half trials of same and half trials of different relationship for each of the three components. *Location Discrimination* was run as a non-speech control task, for which the participants were asked to indicate whether the two sounds were presented at the same or opposite ears, regardless of their speech contents. Stimulus conditions were the same as in *Word Discrimination*.

### Data Collection and Analyses

Four volunteers were excluded from the experiment due to lower accuracy than 85% correct for any task. The remaining 96 participants were randomly assigned to four groups (*N* = 24 per group). Each group was tested with no more than four speech tasks to reduce confusion and fatigue. To compare performance across tasks, a block of 48 trials was collected for each of the six discrimination tasks: Group 1 was tested with location, word, syllable, and tone discrimination, and Group 2 was tested with consonant and vowel discrimination. The order of tasks was randomized across participants. To examine performance variation with stimulus condition within a task, two blocks (96 trials except in one case) were collected for each of the five speech tasks: Group 3 was tested with syllable and tone discrimination, and Group 4 with consonant, vowel, and an extended version of word (140 trials) discrimination. The task order was randomized for the first block and counterbalanced for the second block. Before each task, a 5-trial demo was provided to help the participant understand the task.

For all tasks, performance was measured by error rate and reaction time (RT) on correct trials. RTs shorter than 0.2 s or longer than condition mean by more than two standard deviations, suggesting attention lapse or performance interruption, were excluded (<2% of trials). Mean RT was used in across-task comparisons and median RT was used in within-task analyses to reduce influence of difference in number of trials for different stimulus conditions. Because trial distribution across the eight stimulus conditions was the most skewed in word discrimination (Figure 1; 50% of same-word trials), the 140-trial instead of the 96-trial version was used for within-task analyses to provide at least 10 trials in each stimulus condition. For each across or within task comparison, we started with a full-factorial model of ANOVA including all concerned factors and their interactions, followed by Fisher’s least significant difference (LSD) *post hoc* comparison when needed.

Interference effect between phonemes and lexical tone was indexed by difference in error rate and RT between trials in which the non-target dimension was fixed and trials in which the non-target dimension was varied. There were six interference effects: interference of consonant with tone discrimination (C2T), interference of tone with consonant discrimination (T2C), interference of vowel with tone discrimination (V2T), interference of tone with vowel discrimination (T2V), interference of consonant with vowel discrimination (C2V), and interference of vowel with consonant discrimination (V2C). For example, C2T was the difference between tone discrimination trials in which the two stimuli had the same and different consonants. These trials were matched in terms of the third dimension (vowel), because the three dimensions were independently manipulated for these tasks.

## Results

### Comparison Across Tasks

**Figure 2:**
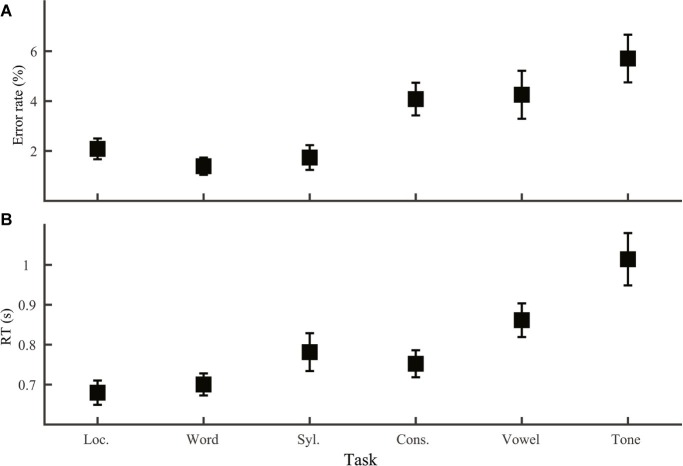
Error rate **(A)** and RT **(B)** across tasks. Error bars were S.E.M.s in this and all the following figures. Acronyms: Loc., Location; Syl., Syllable; Cons., Consonant.

We first compared mean performance across the six discrimination tasks (Group 1 and 2; Figure 2), which allowed us to test both the first prediction regarding comparison between lexical tone and phoneme and the second set of predictions regarding comparison across processing levels. Both error rate (one-way ANOVA, effect of task: *F*_5,138_ = 5.90, *p* < 0.001) and RT (*F*_5,138_ = 8.23, p < 0.001) varied significantly across tasks. LSD *post hoc* comparisons indicate that based on error rate, the six tasks fell into two groups. Location, word, and syllable discrimination yielded the least errors (*p* > 0.40 for comparisons among these three tasks; *p* < 0.04 for comparisons with the other tasks). Consonant, vowel, and tone discrimination were more erroneous (*p* > 0.1 for comparisons among these three tasks; *p* < 0.05 for comparison with the other tasks). RT on correct trials, however, divided the six tasks into three groups. Tone discrimination was the slowest (*p* < 0.05 for comparison with the other tasks), followed by vowel discrimination, which was faster than tone discrimination (*p* = 0.013), slower than location and word discrimination (*p* < 0.01), and did not differ from syllable or consonant (*p* > 0.05) discrimination. The third group included location, word, syllable, and consonant discrimination, which did not differ from each other (*p* ≥ 0.1).

### Word Judgment With Lexical Tone and Segmental Difference

**Figure 3:**
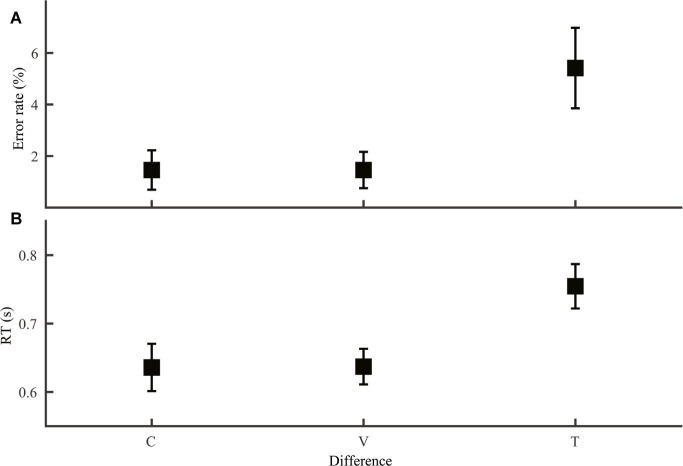
Word discrimination error rate **(A)** and RT **(B)** with difference only in consonant (C), vowel (V) or tone (T).

Our second prediction regarding comparison between lexical tone and phoneme was that same/different word judgment, a phonological decision required in homophone and lexical decision tasks should be harder when the two stimuli differed only in lexical tone than when they differed only in segmental difference. To test this prediction, we compared word discrimination trials with only lexical tone difference and those with only consonant or vowel difference (Group 4; Figure 3). Both error rate (Figure 3A; *F*_2,69_ = 4.45, *p* = 0.015) and RT (Figure 3B; *F*_2,69_ = 4.76, *p* = 0.012) varied with type of difference. LSD *post hoc* pairwise comparisons revealed poorer performance for lexical tone difference than consonant (error rate: *p* = 0.012; RT: *p* = 0.009) or vowel (error rate: *p* = 0.012; RT: *p* = 0.010) difference, but similar performance for the two types of phoneme difference (error rate: *p* = 1.0; RT: *p* = 0.98).

### Interference Between Lexical Tone and Phoneme

**Figure 4:**
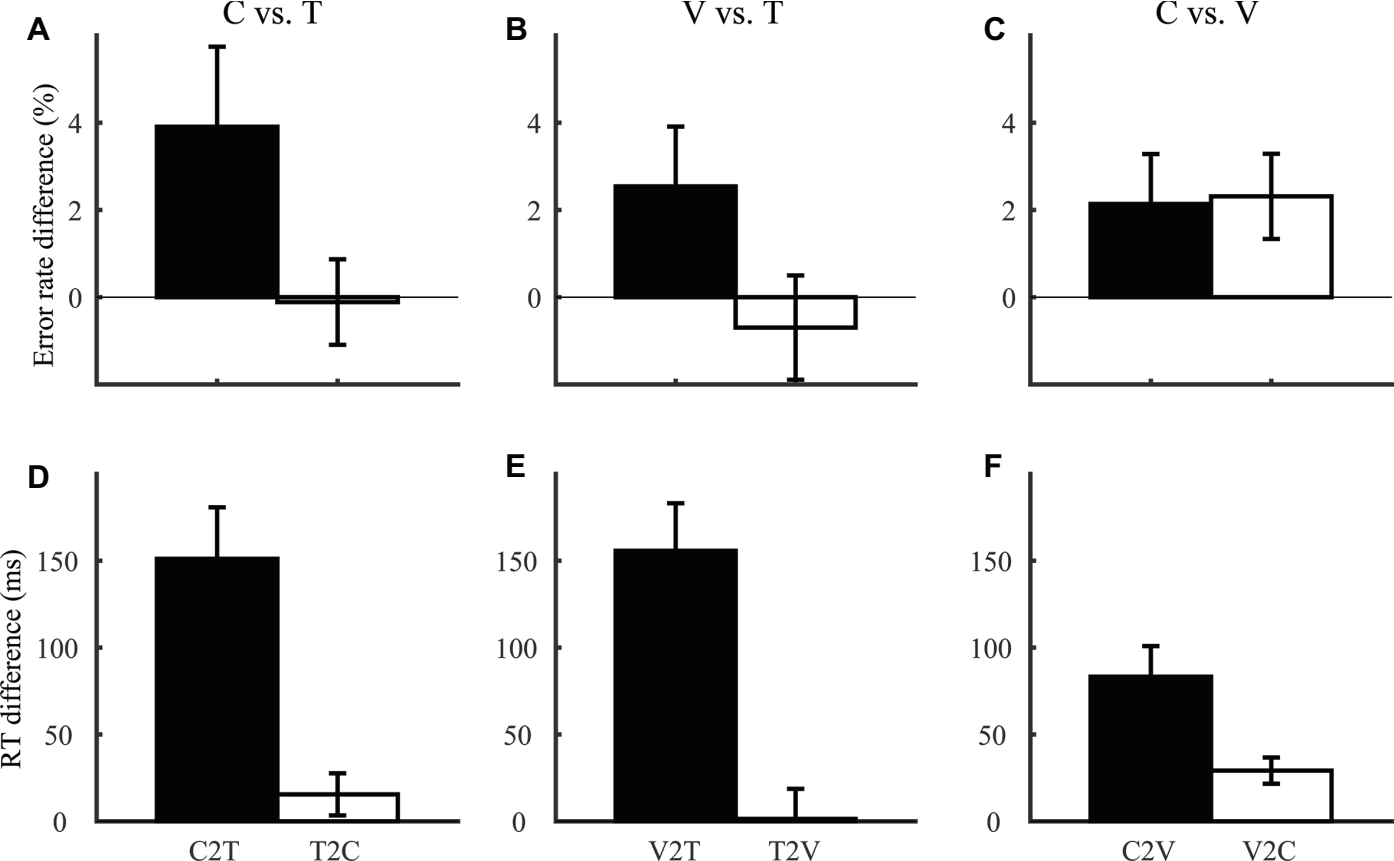
Interference between consonant (C), vowel (V), and tone (T) dimensions, indexed by error rate **(A–C)** and RT **(D–F)** differences. Each effect was labeled in the format of Y2X, indicating interference of the non-target dimension Y with judgments along the target dimension X. For example, C2T referred to interference of consonant variation with tone discrimination, calculated as error rate and RT differences between tone discrimination trials with different and same consonants.

Our third prediction regarding comparison between lexical tone and phoneme was that interference of lexical tone variation with segmental judgments should be smaller than interference in the reverse direction. Consistent with this prediction, for each of tone, consonant, and vowel discrimination (Group 1 and 2; Figure 4), we calculated interference of variation in non-target dimensions (e.g., consonant and vowel) on judgments along the target dimension (e.g., lexical tone). In terms of error rate (Figures 4A–C), there was no significant interference between any pair of dimensions (*F*_1,46_ ≤ 3.73, *p* > 0.05). In terms of RT (Figures 4D–F), the asymmetry in interferences among lexical tone, consonant, and vowel were more salient: C2T was larger than T2C (*F*_1,46_ = 13.6, *p* = 0.001), V2T larger than T2V (*F*_1,46_ = 18.2, *p* < 0.001), and C2V larger than V2C (*F*_1,46_ = 7.78, *p* = 0.008).

To sum up, we confirmed all of the three predictions regarding phoneme-tone comparisons using the same context-free speeded discrimination paradigm. These results were consistent with previous observations of segmental advantage over lexical tone from various tasks and studies (Taft and Chen, 1992; Cutler and Chen, 1997; Ye and Connine, 1999; Tong et al., 2008). Performance across processing levels, however, was in sharp contrast with our expectation.

First, word judgments were more accurate and faster than judgments of phonemes and tones. According to TRACE, representation at each level is available for perceptual decision as soon as it is formed. A paradigm entirely devoid of contextual information such as the current study allows no chance of predicting higher-level representation before its component is extracted or correcting it in cases of erroneous component representations. That is, processing time or error rate of word judgments could not possibly be faster or lower than those of component-level judgments. The exact opposite observation led us to suspect that accessing and initial extraction of phonological information are separated and that somehow word representations are more readily accessible than their components.

Second, atonal syllable judgments were more accurate than the two phoneme tasks and were as fast as consonant discrimination. Applying the TRACE with an addition of tone level to the current study dictates that atonal syllable judgments be done by comparing both consonant and vowel representations. If so, errors of both consonant and vowel representations would manifest in the syllable task, making it less accurate than comparison of single phonemes. If decisions were made based only on consonant representations, as the similar reaction time suggested, accuracy would be severely compromised. The accurate and quick performance of the syllable task in relation to the phoneme tasks leads us to suspect that segmental information is integrated before tone, forming an intermediate representation of atonal syllable before formation of word representation.

Further, performance on word and syllable discrimination was similar to that on location discrimination, a task that did not require speech information, leading us to suspect that speech information required for word and syllable judgments are maintained in a readily accessible state without need of further speech processing.

## Theory

### The Reverse Accessing Model

**Figure 5:**
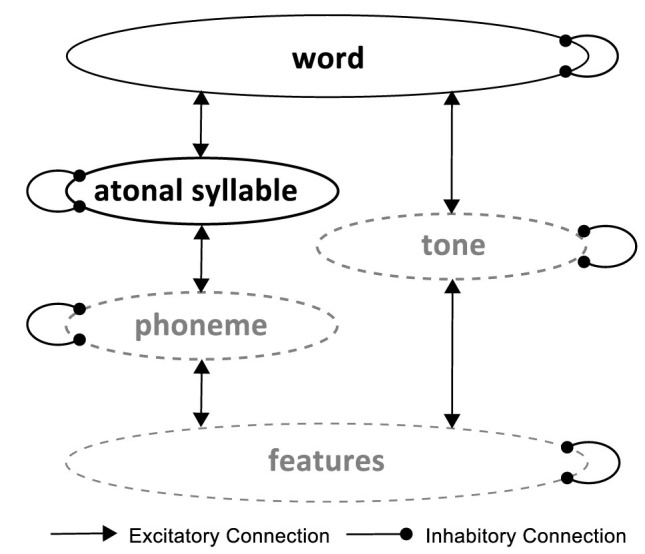
The Reverse Accessing Model (RAM) for sub-lexical phonological processing of tonal speech. Each eclipse indicates a level of processing devoted to a specific type of phonological representation. The solid lines indicate ready access to the extracted information, while the dotted lines mark “hidden” information that can only be accessed by reactivating the system with the perceived word. Information accessing starts from the top and proceeds to the lower level if necessary.

The preceding results, specifically the across-task performance pattern, prompted us to propose a modification of the classic TRACE model (McClelland and Elman, 1986) for sub-lexical phonological processing of Chinese (Figure 5), which we will refer to as the Reverse Accessing Model (RAM). The TRACE consists of three levels of phonological processing: feature (such as acuteness and diffuseness), phoneme, and word, with bidirectional inhibitory connections within a level and excitatory connections between levels. The RAM includes two additional levels specialized for tonal speech: a lexical tone level representing lexical tone information extracted from acoustic features, and a syllable level representing atonal syllable (combination of consonant and vowel segments) before integration of lexical tone. In this way, the RAM allows representations of atonal syllables to be available even before lexical tone is extracted. Further, unlike the TRACE, which subserves working memory as well as perception by maintaining information from all levels at all time points (like a series of memory traces), we assume that information accessing starts from the top and proceeds to the next level if and only if information at the higher level is insufficient for the task at hand. Only information at the syllable level and up is readily accessible. Information at lower levels such as phonemes or lexical tones are “hidden,” in that they can only be accessed for mental operations *via* reactivation of the system by a mental replay of the perceived word. Computationally, the different states of accessibility can be realized by having both time-invariant and time-specific coding in the model [e.g., (Hannagan et al., 2013)]. Functionally, ready accessibility requires maintaining information in working memory, which has limited storage capacity and displays interference among simultaneously maintained information (Baddeley, 2003; Oberauer et al., 2013). Compared to the TRACE, the demand for working memory is markedly reduced in the RAM. It is particularly so for natural speech, in which there are often plenty of contextual information for word representations to be formed or disambiguated without the need to reactivate syllable, tone or phoneme representations, rendering speech perception rely preferentially, in terms of both time order and total contribution, on higher level than lower level processing. The model thus echoes neurophysiological evidence of ultra-rapid cortical activation of words at ~50 ms after required acoustic information (MacGregor et al., 2012), faster than typically observed for phonological processing of lexical tone in passive listening (Yu et al., 2014; Tang et al., 2016) or lexical tasks (Li et al., 2008).

The dual-access mode of the RAM provides a simple account for the across-level performance pattern as well as the asymmetric interference between lexical tone and phoneme. Mode of information accessing divides the speech tasks into two groups: tasks that reply on readily accessible information (word and syllable discrimination), and tasks that require reactivation of hidden information (consonant, vowel, and tone discrimination). As reactivation may introduce additional errors and cost additional time, the RAM predicts lower error rate and smaller reaction time for the ready-access group than the reactivation group, consistent with behavioral observations. If information at all levels were maintained over time, as assumed by the TRACE, one would expect increasingly larger error rate and RT from the phoneme level up to the word level, as error and time cost accumulates in processing. For example, if the consonant /b/ was misrepresented as /d/ at the phoneme level, this error would be passed up to the syllable and word level (and could not be corrected without limit of context or existing vocabulary).

The dual-access mode could also give rise to the asymmetric interference between lexical tone and phoneme observed in the current and previous studies (Repp and Lin, 1990; Tong et al., 2008). A known feature of working memory is that mental operations on one particular memory item are subject to interference from other items (Baddeley, 2003; Oberauer et al., 2013). In the RAM, variations in consonant/vowel are also represented as variations in syllable and would thus be present during lexical tone judgment and influence decision. In contrast, lexical tone information would remain “hidden” during phoneme judgments and have little influence on performance.

The RAM offers specific mechanisms for lexical tone disadvantage in phonological decisions. Consistent with previous studies (Taft and Chen, 1992; Cutler and Chen, 1997; Ye and Connine, 1999), we observed a tone-to-phoneme disadvantage both in word discrimination (Figure 3; better performance with only segmental than with only lexical tone difference) and in across-task comparison (Figure 2; slower tone than consonant and vowel discrimination). According to the RAM, lexical tone disadvantage in the two cases may have different causes. For word discrimination, judgment should be made at the word level, with syllable representations available but phoneme and lexical tone representations not. When the two stimuli differed only in segmental information, word and syllable information would be congruent, both pointing to a “different” response. However, when the two stimuli differed only in lexical tone, syllable information would point to a “same” response, incongruent with word information and interfering with word judgment. The RAM thus attributes lexical tone disadvantage in lexical-level decisions such as homophone and lexical decision tasks to interference from syllable information. Comparison of phoneme and lexical tone discrimination is more complicated, because these tasks can be performed on readily accessible information in some stimulus conditions and require reactivation of lower-level information in others. Difference among these tasks could thus arise from difference in reactivation, difference in mental operations on accessible information, or a combination of both.

The RAM is compatible with the neural imaging evidence suggesting a multi-level cortical network for tone processing (Roll et al., 2015; Shuai and Malins, 2016; Si et al., 2017; Soderstrom et al., 2017; Schremm et al., 2018) and offers a mechanism for the suggested network dynamics to unfold. The top-down reactivation of the phonological network provides a simple mechanism for top-down factors to interact with bottom-up processing (Shuai and Gong, 2014). Maintenance of syllable and word level information is consistent with the suggestion that superior temporal gyrus may encode high-level phonological instead of acoustic information (Chang et al., 2010), possibly memory trace of information chunk larger than phonemes (Schremm et al., 2018). That sub-syllabic information could be reactivated after initial processing provides a mechanistic account for the finding that left inferior frontal gyrus activation correlated with neural processing of tone in two separate time windows (Roll et al., 2015).

Overall, the RAM provides a theoretical framework in which bottom up, auditory processing interacts dynamically with top-down, linguistic processing and/or cognitive factors, resulting in activation patterns tailored to the task demand. The model’s first and primary prediction is that processing of the same phonological unit (phoneme, tone, syllable, etc.) should be task dependent. In this respect, the RAM differs categorically from TRACE like models, which maintain full information for each and all tasks. This prediction can be easily tested using behavioral as well as neural imaging measures. Violations of this prediction such as evidence that phoneme or tone processing remains the same regardless of whether the task demands that information for immediate use would defy the core assumption of the model. Another prediction would be that working memory is closely involved in speech perception and its load would vary according to listening environment and task, consistent with cognitive models of speech comprehension (Rönnberg et al., 2008). Evidence of no or constant employment of working memory would support TRACE like models over the RAM. In the following section, we will begin testing the RAM with its specific predictions of performance on each of the phonological discrimination tasks employed here.

### Testing the Reverse Accessing Model

All the three phonological elements of a word (consonant, vowel, and lexical tone) were manipulated in the discrimination tasks, yielding eight stimulus conditions for each task (Figure 1). The RAM generated specific predictions on how each task should be performed under each stimulus condition. Here, we tested these predictions by examining performance patterns across stimulus conditions.

To facilitate reliability of within-task analyses, we collected two blocks of trials for each task (140 trials for word discrimination and 96 trials for other tasks; Group 3 and 4). For all of the discrimination tasks, the first stimulus of each trial was randomly selected out of a stimulus pool of eight spoken monosyllable words and the second stimulus was determined by stimulus condition. We ruled out effect of character on the first trial (ANOVA, error rate: *F*_7,184_ < 1.73, *p* > 0.1; RT: *F*_7,184_ < 0.73, *p* > 0.1) before we proceeded to examine effect of stimulus condition.

#### Word Discrimination

According to the RAM, word discrimination can be conducted based solely on the readily accessible word information. Syllable information, which is also available, may interfere with task performance when it is incongruent with word information. Information at lower levels would not be reactivated. Therefore, performance should not be influenced by lexical tone information or by phoneme information beyond their representation at the syllable level.

To test these predictions, we first examined whether information from the phoneme level had an impact on performance beyond that of the syllable level. Variation at the syllable level could result from variation in consonant, in vowel, or in both at the phoneme level. Among trials with syllable variation, neither error rate nor RT (ANOVA, effect of variation type: *F*_2,69_ ≤ 0.35, *p* > 0.05) differed with the type of variation at the phoneme level, consistent with the prediction that consonant and vowel variations were only collectively represented as syllable variation and information at the phoneme level had no further contribution.

**Figure 6:**
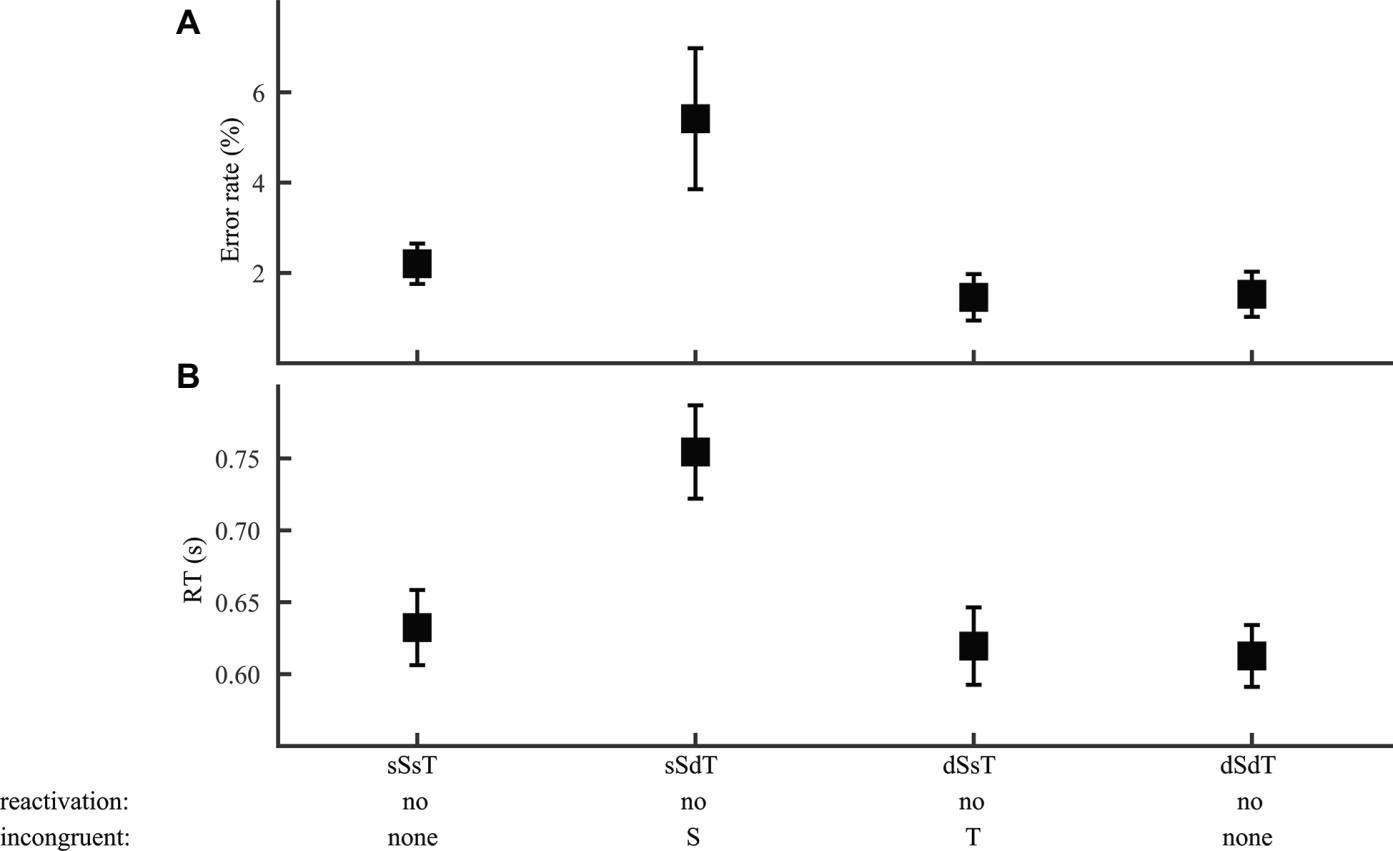
Word discrimination. Error rate **(A)** and RT **(B)** were plotted for conditions differing in syllable and lexical tone manipulations: same-syllable-same-tone (sSsT), same-syllable-different-tone (sSdT), different-syllable-same-tone (dSsT), and different-syllable-different-tone (dSdT). The RAM’s prediction of reactivation of lower-level information and information incongruent with word judgment were marked.

This result allowed us to narrow down to two independent components: syllable and lexical tone (Group 4; Figure 6). Two sources of information were regarded as congruent when they pointed to the same response and as incongruent when they pointed to different responses. Consistent with the RAM’s prediction, error rate (Figure 6A) and RT (Figure 6B) of word discrimination varied significantly with congruence between syllable and word (ANOVA, error rate: *F*_1,93_ = 10.8, *p* = 0.001; RT: *F*_1,93_ = 16.0, *p* < 0.001), but not with congruence between lexical tone and word (error rate: *F*_1,93_ = 0.14, *p* = 0.71; RT: *F*_1,93_ = 0.008, *p* = 0.93).

#### Syllable Discrimination

Given the top-down accessing order assumed by the RAM, syllable judgments should be made at the word level when the two stimuli were of the same word and at the syllable level after disregarding word information in the remaining cases. The extra search required by different-word stimuli may lead to a behavioral disadvantage. Unlike word discrimination, where syllable information may interfere with word judgment, we expect no interference of word information with syllable judgment, as word information would have been searched and disregarded when syllable information is evaluated. Lexical tone and phoneme information should not be reactivated, with no interference from incongruent lexical tone information or influence of phoneme information beyond the syllable level.

**Figure 7:**
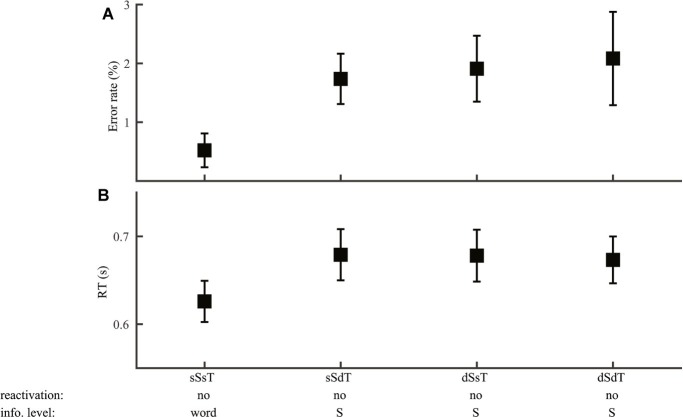
Syllable discrimination. Error rate **(A)** and RT **(B)** were plotted for conditions with different syllable and lexical tone manipulations: same-syllable-same-tone (sSsT), same-syllable-different-tone (sSdT), different-syllable-same-tone (dSsT), and different-syllable-different-tone (dSdT). The RAM’s prediction of reactivation of lower-level information and the information level for syllable judgments were marked.

Consistent with the prediction of no phoneme reactivation, syllable discrimination performance (Group 3) did not differ with the type of phoneme variation (ER: *F*_2,69_ = 1.79, *p* = 0.18; RT: *F*_2,69_ = 1.61, *p* = 0.21), allowing us to examine segmental influence at the syllable level only (Figure 7). Neither lexical tone variation nor congruence of lexical tone with syllable had any impact (error rate: *F*_1,46_ ≤ 1.2, *p* > 0.05; RT: *F*_1,46_ ≤ 0.86, *p* > 0.05), supporting no reactivation of lexical tone information. As syllable discrimination performance did not differ among the three different-word conditions (Figure 7A, error rate: *F*_2,69_ = 0.012, *p* = 0.99; Figure 7B, RT: *F*_2,69_ = 0.80, *p* = 0.92), trials on these conditions were pooled together to compare with same-word (same-syllable-same-tone) trials. Consistent with the prediction that different-word judgment involved additional information accessing steps than same-word judgment, error rate (*F*_1,46_ = 6.48, *p* = 0.014) was smaller in same-word than different-word trials, though RT did not differ between the two cases (*F*_1,46_ = 2.26, *p* = 0.14).

#### Tone Discrimination

Applying the RAM to tone discrimination yielded a more complex pattern of performance. First, for same-word stimuli, lexical tone can be inferred solely from word information. Performance in this condition should be both accurate and quick. Second, for different-word stimuli with the same syllable, lexical tone can also be inferred by comparing word and syllable information. Performance in this condition should be accurate (because reactivation is not needed) but slower (because it takes time to deduce lexical tone relation from word and syllable information). Only for stimuli with different syllables must information from the lexical tone level be reactivated for task performance. In these cases, same-tone responses would be harder than different-tone responses due to interference from incongruent information at the syllable and word levels.

**Figure 8:**
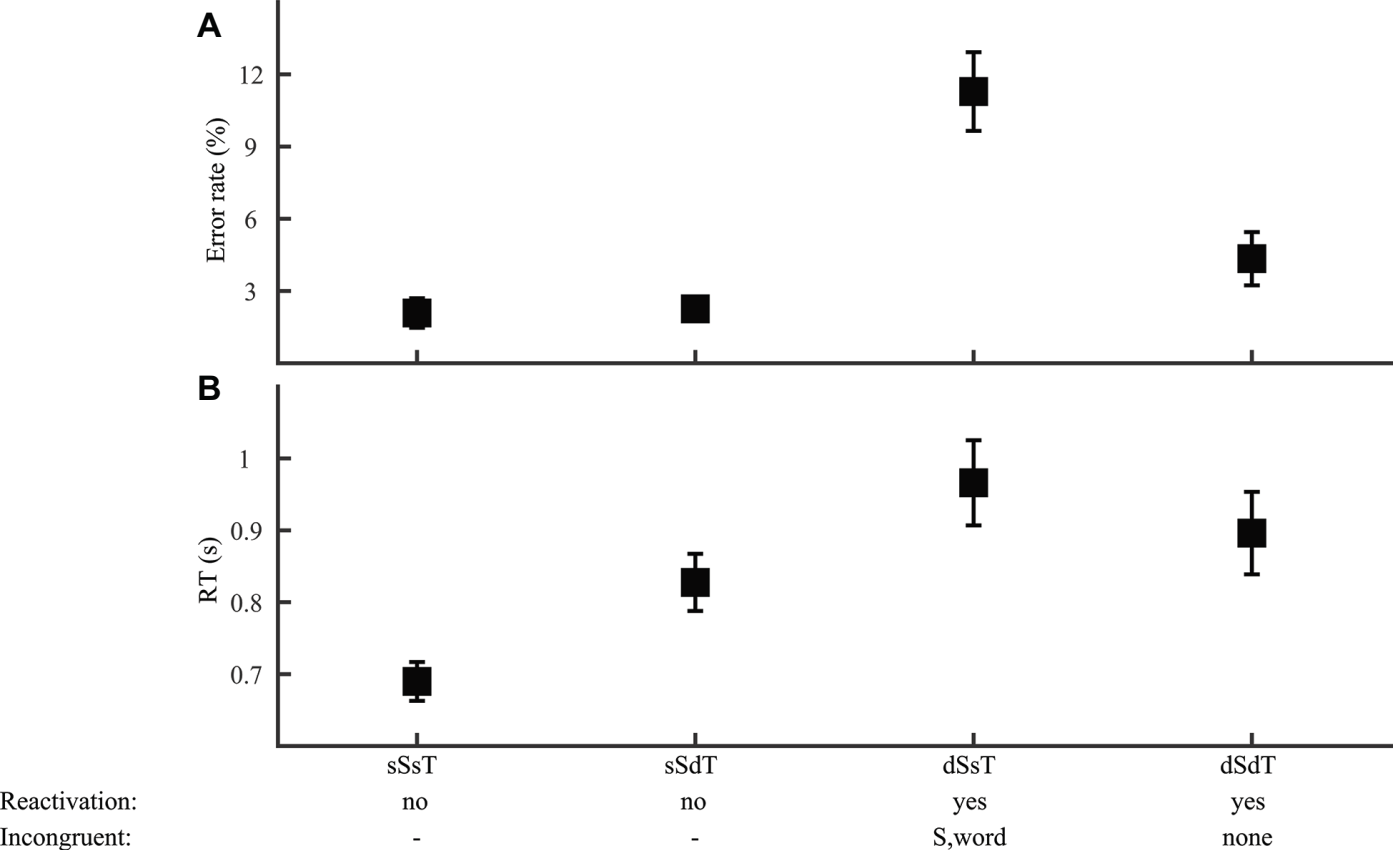
Tone discrimination. Error rate **(A)** and RT **(B)** were plotted for conditions with different syllable and lexical tone manipulations: same-syllable-same-tone (sSsT), same-syllable-different-tone (sSdT), different-syllable-same-tone (dSsT), and different-syllable-different-tone (dSdT). The RAM’s predictions regarding lexical tone reactivation and incongruent information for reactivation conditions were marked.

For tone discrimination (Group 3), we also first ruled out contribution of the phoneme level, as neither error rate nor RT (*F*_2,69_ ≤ 0.17, *p* > 0.05) differed with the type of phoneme variation (consonant, vowel, or both). This result allowed us to focus on syllable and lexical tone manipulations (Figure 8). Consistent with the prediction that lexical tone was reactivated only when the stimuli had different syllables, performance was more erroneous (Figure 8A; syllable by lexical tone ANOVA, effect of syllable: *F*_1,92_ = 28.1, *p* < 0.001) and slower (Figure 8B; *F*_1,92_ = 13.0, *p* < 0.001) in different-syllable than in same-syllable conditions. An interaction between syllable and lexical tone (error rate: *F*_1,92_ = 11.2, *p* = 0.001; RT: *F*_1,92_ = 4.73, *p* = 0.032) indicated different impacts of lexical tone manipulation for each type of syllable manipulation. Planned comparisons revealed that, for same-syllable (no-reactivation) conditions (Figure 8, sSsT and sSdT), different-tone responses were equally accurate as (*F*_1,46_ = 0.048, *p* = 0.83) but slower than (*F*_1,46_ = 5.66, *p* = 0.001) same-tone responses. This result was consistent with the prediction that lexical tone judgment was based on word information when the stimuli were of the same word (sSsT) but was deduced by comparing word and syllable information when the stimuli were different words with the same syllable (sSdT). The different-syllable (reactivation) conditions (Figure 8, dSsT and dSdT) had similar RT (*F*_1,46_ = 0.72, *p* = 0.40), but same-tone responses (dSsT) were more erroneous (*F*_1,46_ = 12.4, *p* = 0.001) than different-tone responses (dSdT), possibly reflecting interference of incongruent syllable information with lexical tone judgment.

#### Consonant and Vowel Discrimination

Similar to tone discrimination, consonant and vowel discrimination is also predicted by the RAM to depend on reactivation and congruence between existing information. First, in all conditions, lexical tone information is irrelevant and should not be reactivated. Second, in same-syllable conditions, phoneme judgments could be made based on readily accessible information and require no reactivation of phoneme representations. Same-word stimuli (word-level decisions) might have a slight advantage over different-word stimuli (syllable-level decisions) due to shorter search route, as shown for syllable discrimination. However, compared to performance variation caused by reactivation or interference, this advantage should be of small magnitude and may not be detectable. Third, in different-syllable conditions, decisions require reactivation of phoneme representations and should be subject to interference from incongruent syllable (and possibly word) information. Consonant and vowel information are represented at the same level but are temporally separated in reactivation. Depending on the extent of temporal separation, there may be interference between the phonemes, particularly when the non-target phoneme is activated earlier than the target phoneme (in the case of consonant interference with vowel discrimination).

**Figure 9:**
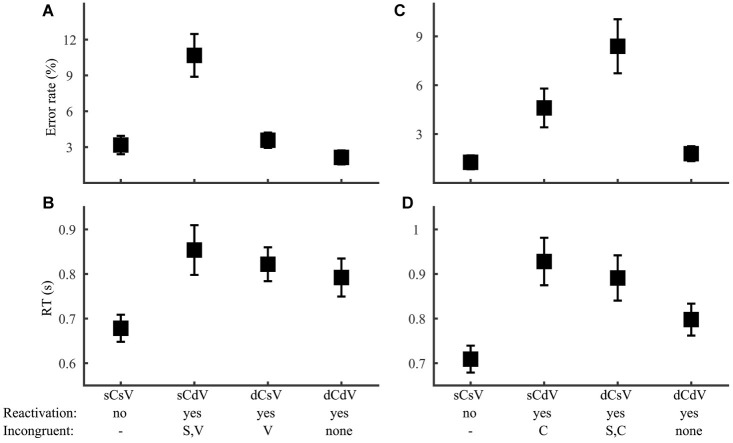
Consonant and vowel discrimination. Error rate **(A,C)** and RT **(B,D)** for consonant **(A,B)** and vowel **(C,D)** discrimination were plotted for conditions with different phoneme manipulations: same-consonant-same-vowel (sCsV), same-consonant-different-vowel (sCdV), different-consonant-same-vowel (dCsV), and different-consonant-different-vowel (dSdT). The RAM’s predictions regarding phoneme reactivation and incongruent information for reactivation conditions were marked.

For consonant and vowel discrimination (Group 4), we first tested the prediction of no reactivation of lexical tone. Consistent with this prediction, performance did not vary with either lexical tone variation or congruence of lexical tone with target phoneme (consonant discrimination error rate: *F*_1,46_ ≤ 1.82, *p* > 0.05; RT: *F*_1,46_ ≤ 0.069, *p* > 0.05; vowel discrimination error rate: *F*_1,44_ ≤ 0.68, *p* > 0.05; RT: *F*_1,44_ ≤ 0.12, *p* > 0.05). Specifically, there was no difference between the two same-syllable conditions (consonant discrimination error rate: *F*_1,46_ = 0.007, *p* = 0.93; RT: *F*_1,46_ = 0.45, *p* = 0.51; vowel discrimination error rate: *F*_1,46_ = 0.49, *p* = 0.48; RT: *F*_1,46_ = 0.24, *p* = 0.62), indicating similar performance with word-level and syllable-level decisions. The lack of lexical tone influence allowed us to focus on consonant and vowel manipulations (Figure 9). Consistent with the reactivation assumption, performance was better in same-syllable than different-syllable trials for both consonant (error rate: *F*_1,46_ = 4.33, *p* = 0.043; RT: *F*_1,46_ = 6.55, *p* = 0.014) and vowel (error rate: *F*_1,46_ = 17.0, *p* < 0.001; RT: *F*_1,46_ = 8.76, *p* = 0.005) discrimination. Further, among the three reactivation conditions (Figure 9; sCdV, dCsV, and dCdV), we simultaneously examined interference from syllable and non-target phoneme. For consonant discrimination, error rate (Figure 9A) varied significantly with syllable (*F*_1,69_ = 19.1, *p* < 0.001), but not with vowel (*F*_1,69_ = 0.78, *p* = 0.38) congruence, while RT (Figure 9B) did not show any interference effect (syllable congruence: *F*_1,69_ = 0.24, *p* = 0.63; vowel congruence: *F*_1,69_ = 0.89, *p* = 0.35). Vowel discrimination showed the same pattern, with syllable (Figure 9C; *F*_1,69_ = 4.91, *p* = 0.030) but not consonant (*F*_1,69_ = 2.71, *p* = 0.11) interference on error rate and no interference effect on RT (Figure 9D; syllable congruence: *F*_1,69_ = 0.31, *p* = 0.58; consonant congruence: *F*_1,69_ = 1.95, *p* = 0.17). The presence of syllable interference for both phoneme tasks was consistent with the RAM’s assumption. The lack of interference between phonemes, particularly in vowel discrimination, supports complete temporal separation of consonant and vowel in phoneme reactivation.

#### Word- and Reactivated Information-Based Decisions Across Tasks

**Figure 10:**
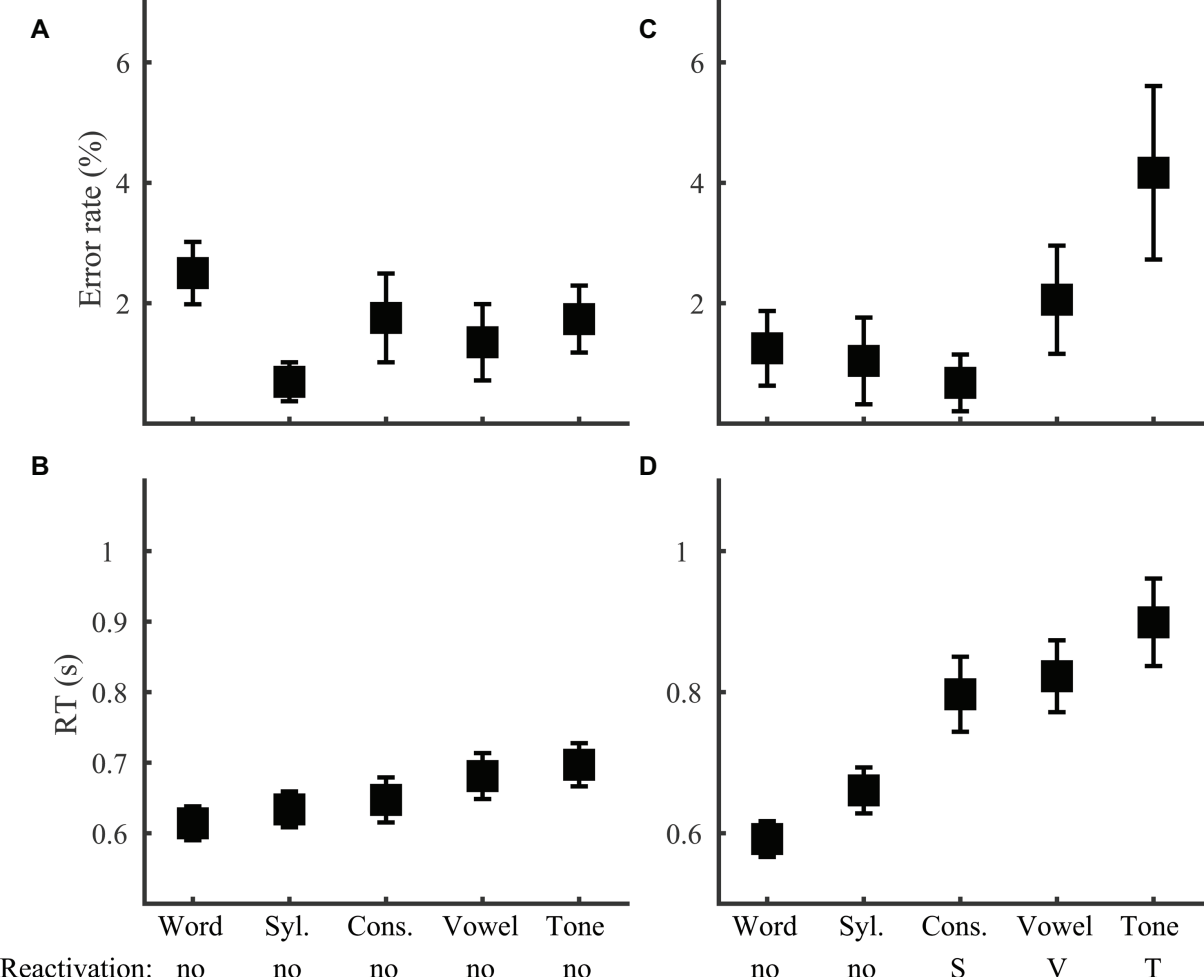
Condition comparison across tasks. Error rate **(A,C)** and RT **(B,D)** in the same-consonant-same-vowel-same-tone [sCsVsT; **(A)**, b] and different-consonant-different-vowel-different-tone [dCdVdT; **(C)**, d] conditions were plotted for the five speech tasks. Acronyms: Syl., Syllable; Cons., Consonant.

The RAM predicts interference between incongruent information, which we have shown to occur in different conditions for different types of phonological judgment. In two of the stimulus conditions (same-consonant-same-vowel-same-tone, sCsVsT and different-consonant-different-vowel-different-tone, dCdVdT), however, information from all levels are congruent, allowing us to compare phonological decisions across tasks without the complication of interference. According to the RAM, all types of phonological decisions in the sCsVsT (same-word) condition should be made on the word level. Consistent with this prediction, neither error rate (Figure 10A; *F*_4,115_ = 1.33, *p* = 0.26) nor RT (Figure 10B; *F*_4,115_ = 1.37, *p* = 0.25) of same-word trials varied across tasks. In the dCdVdT condition, word and syllable judgments should be based on readily accessible information, while phoneme and lexical tone judgments should depend on reactivated lower-level information. The dCdVdT condition thus provides an opportunity to directly compare reactivation of phoneme and lexical tone. Interestingly, RT did not vary among the three reactivation tasks (Figure 10D; *F*_2,69_ = 0.91, *p* = 0.41) and error rate only showed a trend of variation (Figure 10C; *F*_2,69_ = 2.98, *p* = 0.057) disfavoring tone discrimination. The result suggests that reactivating lexical tone is probably not harder than reactivating phoneme. Comparing with overall performance across the phoneme and lexical tone tasks (Figure 2), it appears that while the pattern of error rate could be driven by the reactivation process, the difference in RT was clearly not. Alternatively, RT difference should result from different mental operations on accessible information, notably in the same-syllable-different-tone (sCsVdT) condition, in which phoneme judgments could be inferred directly from syllable information, but lexical tone judgment required comparing word and syllable information. Supporting this idea, RT in the sCsVdT condition was significantly larger (*F*_2,69_ = 6.33, *p* = 0.003) in tone than in consonant (LSD *post hoc* comparison: *p* = 0.001) and vowel (*p* = 0.008) discrimination.

## Discussion

In the current study, we proposed and tested a general framework for sub-lexical phonological processing of tonal languages. We first unitedly tested a variety of expectations based on the existing literature and speech perception models using the same speeded discrimination paradigm. While the results generally confirmed the phoneme-over-tone advantage under context-free situations in previous reports (Taft and Chen, 1992; Cutler and Chen, 1997; Ye and Connine, 1999), they also revealed an advantage for word and atonal syllable judgments over phoneme and lexical tone judgments that challenged the current understanding of phonological processing. Inspired by this finding, we proposed a Reverse Accessing Model (RAM) of sub-lexical phonological processing for tonal speech and subsequently confirmed its predictions on how various types of phonological judgments should be made under different stimulus conditions.

The RAM was constructed after the renowned TRACE model (McClelland and Elman, 1986) for non-tonal speech perception. To our awareness, the only existing speech perception model for tonal languages is an extension of the TRACE proposed by Ye and Connine (1999), in which lexical tone is represented at a separate “toneme” level in parallel to phoneme (as implied by the term “toneme”). The RAM differs from the TRACE and the “toneme” proposal in two key aspects. First, a level of representation is introduced for atonal syllable, allowing consonant and vowel information to integrate even before lexical tone is extracted. Second, information is accessed in the reverse order of information processing, starting from the top level and proceeding to lower levels upon task demand. Further, only information at the syllable level and above (the word level) are readily accessible, presumably by being maintained in working memory, while information at phoneme and lexical tone levels can only be retrieved by reactivating the system with a mental replay of the perceived word. The RAM has several features that make it theoretically and practically accommodating. First, the dual-access mode sets a limit to the amount of readily accessible information, considerably reducing the demand for computational resources and working memory capacity. In doing so, low-level information such as phoneme or tone could be reactivated from higher-level representations after initial extraction, consistent with the two temporal windows of top-down and bottom-up interactions observed in ERP (Shuai and Gong, 2014) and MRI (Roll et al., 2015) studies. Second, the RAM takes into consideration interference among competing information, a known phenomenon in the literature of working memory and decision making. This feature allowed the RAM to unitedly account for studies of between-dimension interference and studies of lexical/phonological decisions. Third, syllable representation is the earliest and smallest unit of phonological information immediately available for mental operations. This feature concurs with the proposal of atonal syllable as the “approximate unit,” i.e., the first level of phonological preparation during speech production (O’Seaghdha et al., 2010; Chen and Chen, 2013). That atonal syllable may be the first functionally critical level of phonological representation for both perception and production of tonal languages supports the idea that speech perception and production are closely and deeply connected (for review, see Casserly and Pisoni, 2010). Fourth, the architecture of the RAM implies an advantage of lexical tone over phoneme in top-down feedback, in that the lexical tone level is one connection closer to the word level than the phoneme level. As each connection adds delay and noise, the shorter top-down transmission distance could translate into greater contextual influence on the lexical tone than on the phoneme level. The RAM is thus compatible with observations that the lexical tone disadvantage can be removed (Liu and Samuel, 2007; Malins and Joanisse, 2010) or even reversed (Ye and Connine, 1999) by a constraining context, a phenomenon (Ye and Connine, 1999) aimed to explain in their “toneme” proposal by assuming stronger top-down feedback to lexical tone than phoneme.

While we experimentally confirmed multiple lines of previous evidence for a relative weaker role of lexical tone than segmental information in speech perception, the RAM shed new lights to the nature of the lexical tone disadvantage. One line of evidence for lexical tone disadvantage is that lexical-decision tasks such as real/non-real word or homophonic judgments were harder with lexical tone than segmental differences (Taft and Chen, 1992; Cutler and Chen, 1997). Using the speeded discrimination paradigm, we observed a similar disadvantage for tone- than phoneme-based word judgment (Figure 3). Such results have been taken as evidence for difficulty in accessing lexical tone relative to segmental information (Taft and Chen, 1992; Cutler and Chen, 1997) or reflection of lower priority of lexical tone information in lexical selection (Wiener and Turnbull, 2016). However, it has been suggested that differential impacts of segmental and supra-segmental information should only be observed for tasks focusing on sub-lexical levels, not for tasks conducted at or beyond the lexical level (Soto-Faraco et al., 2001). At the lexical level, segmental and lexical tone information would have already been integrated, and contributions of individual phonological elements should be indistinguishable. The RAM reconciles the conflict by attributing the apparent lexical tone disadvantage to interference of incongruent syllable information with word-level judgments. Similar interpretations may apply to results obtained by manipulating individual phonological components in other word- or higher-level tasks (e.g., Brownschmidt and Cansecogonzalez, 2004; Schirmer et al., 2005; Malins and Joanisse, 2012).

A separate line of evidence for lexical tone disadvantage is that lexical tone judgment is more vulnerable to interference from segmental variation than vice versa (Repp and Lin, 1990; Tong et al., 2008), which we also confirmed in the current paradigm (Figure 4). Interference between two dimensions of a stimulus has conventionally been taken to indicate integration of the dimensions, in that information in one dimension cannot be separated from that in the other (Melara and Marks, 1990). Asymmetric interference, or asymmetric integrality, between lexical tone and phoneme has been suggested to reflect greater salience of phoneme than lexical tone information (Tong et al., 2008). In the RAM, asymmetric interference arises from asymmetry in information processing circuits: phoneme variations are also represented as readily accessible syllable information, which is present during lexical tone judgment and may influence performance, while lexical tone information remains hidden during phoneme judgment and has little impact. The RAM thus lends a mechanistic definition to dimensional integrality and information salience in phonological decisions. Further, the asymmetric interference favoring vowel over lexical tone has been shown to pertain to tonal language experiences (Repp and Lin, 1990). This finding appears counterintuitive at the first glimpse, as lexical tone information is more pervasive and important in tonal than non-tonal languages. However, the language dependence is consistent with the RAM’s account. The asymmetry between lexical tone and phoneme processing in the RAM is caused by the presence of the syllable level, which is introduced as a process specialized for tonal languages. The absence of syllable level in speakers of non-tonal languages removes the asymmetry of the phonological processing network and hence asymmetric interference between phoneme and lexical tone.

A third line of evidence for lexical tone disadvantage involved comparison of judgments on individual phonological components. For example, in a word reconstruction task requiring turning a non-word into a real word by altering a single phonological element, performance was more accurate and faster for altering lexical tone than altering consonant or vowel, and lexical tone was preferentially altered when the element to change was not specified (Wiener and Turnbull, 2016). These results, taken to reflect lower priority of lexical tone than phoneme in constraining lexical selection (Wiener and Turnbull, 2016), are predicted by the RAM. According to the RAM, reconstruction can be done with only readily accessible information by activating representations of existing words associated with the syllable of the non-word. For instance, the non-word /su3/ should be represented as /su/ at the syllable level, which should be connected with real-word representations /su1/, /su2/, and /su4/ at the word level. Selecting such a real-word representation results in apparent lexical tone alteration and should be the preferred option in free reconstruction. In contrast, altering consonant or vowel would require reactivation of lower-level information. For instance, turning /su3/ to /tu3/ would require reactivating the vowel /u/ and the lexical tone /3/ and then activating existing words associated with the reactivated representations. In this case, the poorer performance for phoneme than lexical tone alteration results from the additional cost of reactivation. An instance of direct comparison between lexical tone and phoneme reactivation was possibly offered in the report of Ye and Connine (1999) that while monitoring for a combination of vowel and lexical tone, detecting a vowel mismatch was faster than detecting a lexical tone mismatch. In the current study, tone discrimination was slower than consonant and vowel discrimination (Figure 2). This result bore surface consistence with that of Ye and Connine (1999), which they took to indicate later availability of lexical tone than vowel information. However, in the speeded discrimination paradigm, a closer examination revealed that lexical tone reactivation was no slower than phoneme reactivation (Figure 10). Rather, lexical tone judgment was slower than phoneme judgment in a stimulus condition that did not require reactivation, due to more complex mental operations on readily accessible information. Was lexical tone monitoring slower than vowel monitoring for similar reasons? Applying the RAM to the syllable monitoring task of Ye and Connine (1999) revealed that, as all phonological components varied across trials, readily accessible information on the word and syllable levels would be insufficient for task performance. Detecting the presence of a vowel-tone combination should reactivate both vowel and lexical tone. However, because the task required two judgments at each trial, an alternative explanation to slower lexical tone than vowel reactivation is that participants tended to make vowel judgment/response before lexical tone judgment/response. To compare lexical tone and vowel reactivation without the complication of dual decisions, lexical tone and vowel monitoring should be conducted on separate trials.

In summary, we have shown in the current study that the RAM provides a simple and congruent account for tonal speech perception from a variety of tasks and perspectives. While further testing and elaborations are needed, the RAM offers a general framework to understand sub-lexical phonological processing of tonal speech and related disorders.

## Data Availability Statement

The datasets generated for this study are available on request to the corresponding author.

## Ethics Statement

The studies involving human participants were reviewed and approved by Beijing Normal University Research Ethics Committee. The patients/participants provided their written informed consent to participate in this study.

## Author Contributions

Y-XZ conceived and designed the study, and prepared the manuscript. XG, T-TY, D-LT, and TH conducted the experiments and analyzed the data. HS and YN reviewed and edited the manuscript.

### Conflict of Interest

The authors declare that the research was conducted in the absence of any commercial or financial relationships that could be construed as a potential conflict of interest.

## Footnotes

^1^www.neospeech.com
